# Optimization and Evaluation of a Chitosan/Hydroxypropyl Methylcellulose Hydrogel Containing Toluidine Blue O for Antimicrobial Photodynamic Inactivation

**DOI:** 10.3390/ijms160920859

**Published:** 2015-09-01

**Authors:** Chueh-Pin Chen, Chien-Ming Hsieh, Tsuimin Tsai, Jen-Chang Yang, Chin-Tin Chen

**Affiliations:** 1 Department of Biochemical Science and Technology, National Taiwan University, Taipei 106, Taiwan; E-Mail: d00b22004@ntu.edu.tw; 2 Department of Cosmetic Science, Providence University, Taichung City 433, Taiwan; E-Mail: chienming.hsieh@gmail.com; 3 Graduate Institute of Biomedical Materials and Tissue Engineering, College of Oral Medicine, Taipei Medical University, Taipei 110, Taiwan; E-Mail: tmtsai00@gmail.com; 4 School of Dental Technology, College of Oral Medicine, Taipei Medical University, Taipei 110, Taiwan; E-Mail: yang820065@tmu.edu.tw

**Keywords:** chitosan, hydrogel, toluidine blue O, photodynamic inactivation

## Abstract

Photodynamic inactivation (PDI) combined with chitosan has been shown as a promising antimicrobial approach. The purpose of this study was to develop a chitosan hydrogel containing hydroxypropyl methylcellulose (HPMC), chitosan and toluidine blue O (TBO) to improve the bactericidal efficacy for topical application in clinics. The PDI efficacy of hydrogel was examined *in vitro* against the biofilms of *Staphylococcus* *aureus* (*S. aureus*) and *Pseudomonas* *aeruginosa (P. aeruginosa*). Confocal scanning laser microscopy (CSLM) was performed to investigate the penetration level of TBO into viable *S*. *aureus* biofilms. Incorporation of HMPC could increase the physicochemical properties of chitosan hydrogel including the hardness, viscosity as well as bioadhesion; however, higher HMPC concentration also resulted in reduced antimicrobial effect. CSLM analysis further demonstrated that higher HPMC concentration constrained TBO diffusion into the biofilm. The incubation of biofilm and hydrogel was further performed at an angle of 90 degrees. After light irradiation, compared to the mixture of TBO and chitosan, the hydrogel treated sample showed increased PDI efficacy indicated that incorporation of HPMC did improve antimicrobial effect. Finally, the bactericidal efficacy could be significantly augmented by prolonged retention of hydrogel in the biofilm as well as in the animal model of rat skin burn wounds after light irradiation.

## 1. Introduction

Microorganisms, such as bacteria and yeast, have been recognized for decades to cause human and animal diseases. Biofilm is the main growth form of microorganisms in nature. It is a non-homogeneous body consisting of microorganisms and extracellular polymer substrate (EPS). This EPS matrix provides structural stability and protection to the biofilm against adverse environmental conditions. Generally, biofilm has a higher resistance to antimicrobial drugs, 500–1000 times higher when compared to the planktonic cells [1]. Consequently, biofilm presents a difficult challenge to clinicians due to their persistent nature, inability to be cultured with standard techniques, and resistance to conventional antimicrobial therapy.

Antimicrobial photodynamic inactivation (PDI) has been developed as an alternative therapeutic tool against bacterial infection and garnering considerable interest in the management of drug-resistant bacterial strains [2,3]. PDI as a bactericide employs a combination of nontoxic photosensitizer (PS) and visible light to generate cytotoxic species. Following light irradiation, the activated PS reacts with molecules in its direct environment, either through electron transfer producing free radicals, which can further interact with oxygen to produce reactive oxygen species (type I reaction) or through energy transfer, generating highly reactive singlet oxygen in the presence of oxygen (type II reaction). PDI was shown to be effective against Gram-positive and Gram-negative bacteria, yeast, as well as antibiotic-resistant strains. Due to the direct binding of PS to the cell wall and membranes, PDI causes direct damage to the cell and no resistance was reported in the bacteria under repeated PDI treatment [4,5].

Previously, we showed that chitosan could potentiate the efficacy of PDI against both Gram-positive and Gram-negative prokaryotic bacteria in planktonic cells and biofilms [6]. In addition, chitosan has been shown to potentiate the efficacy of PDI in planktonic cells and biofilms of *C*. *albicans*. Following PDI, the addition of chitosan greatly augmented the killing of *C*. *albicans* and drug-resistant clinical isolates [7]. These results indicate the combination of PDI and chitosan is quite promising for eradicating microbial infections. For clinical application, it is necessary to develop a suitable delivery system carrying PS and chitosan. In this regards, chitosan hydrogels represent a possible solution for PDI. However, chitosan hydrogels exhibit relatively low mechanical strength and limited ability to control the release of encapsulated compounds [8]. For clinical application, increasing the bioadhesion characteristics would prolong its contact with the treatment site and further increase the release of encapsulated drugs. Hydroxypropyl methylcellulose (HPMC) is an attractive nonionic water-soluble cellulose, due to being a natural polymer and non-toxic. HPMC presents several characteristics such as high swell ability and surface activity [9,10] and is the most widely used polymers in oral drug delivery systems for control release. HPMC provides controlled release once it hydrates to form a gelatinous layer, which controls the water transport in the system. Thus, incorporating HPMC might be a beneficial approach to enhance the mechanical strength of chitosan hydrogel with good retention property, which is expected to provide better therapeutic efficiency.

In the present study, toluidine blue O (TBO) and chitosan were mixed with various amounts of HPMC to form chitosan/HPMC hydrogel (HCT hydrogel). The physical characterization of HCT hydrogel was tested for its viscosity, extrudability, and mucoadhesive properties. The PDI efficacy of HCT hydrogel was further evaluated on the biofilm of *S*. *aureus* and *P*. *aeruginosa*. We hypothesized that such system would provide an intimate contact for longer duration between the drug delivery system and adsorption site. Consequently, the PDI efficacy is significantly improved due to longer retention of hydrogel as possessing stronger mucoadhesive strength. The enhancement and formulation strategies could be helpful in developing a drug delivery system for topical antimicrobial photodynamic therapy.

## 2. Results and Discussion

### 2.1. Formulation Design and Characterization of HCT Hydrogel

In this study, we used HPMC as a gelling agent to develop chitosan hydrogels (named as HCT hydrogel) for PDI. Table 1 shows the composition of HCT hydrogel containing chitosan, TBO, and different concentrations of HPMC. For comparison, TBO with chitosan and TBO alone were used as controls to evaluate the PDI efficacy of HCT hydrogel. These formulations were prepared with an intention to optimize the content of HPMC in HCT hydrogel and understand its influence on the mechanical properties and the antimicrobial effect. Table 2 summarized the physical properties and texture parameters including hardness, adhesion, and compressibility of HTC hydrogel and a marketed topical antibiotic gel (ELYZOL^®^). The viscosity of HCT hydrogel was increased when increasing the content of HPMC (from 0.25% to 3%, *w*/*w*) in HTC hydrogel. The presence of HPMC caused the viscosity significantly increased from 36.56 to 629.8 cps when comparing with those of the controls (4.95 and 4.59 cps). Among the formulations tested, HCT hydrogel (F-3) containing 1% (*w*/*w*) HPMC appeared to be the most comparable formulation to the marketed product (ELYZOL^®^) regarding the viscosity.

Texture analysis provides deeper insight on the hydrogel properties and enables correlation to applicability of the formulation. The amount of gelling agent in a formulation is of great importance with respect to its textural properties. It is expected that hydrogel adhesiveness would be correlated to hydrogel bio-adhesiveness. The results obtained by texture analysis showed the same trend as those found in viscosity test. When we increased HPMC concentration to HCT hydrogel, the hardness, adhesiveness, as well as compressibility for hydrogels were increased. Minimum and maximum hardness, compressibility, and adhesiveness values were exhibited by formulations containing 0.25% and 3% (*w*/*w*) of HPMC, respectively. Similarly, Ferrari *et al.* [11] reported that the strength of HPMC gels increased as the polymer concentration was increased. These results demonstrate that the incorporation of HPMC could enforce the structure of HCT hydrogel. In addition, the F-1 HCT hydrogel containing 0.25% (*w*/*w*) HPMC exhibited relatively similar textural properties as compared to ELYZOL^®^.

**Table 1:** Formulation composition of HCT hydrogel.

Formulation	HPMC ^1^ (%, *w*/*w*)	Chitosan ^2^ (%, *w*/*w*)	TBO (μM)
F-1	0.25	0.25	20
F-2	0.5	0.25	20
F-3	1	0.25	20
F-4	2	0.25	20
F-5	3	0.25	20

^1^ HPMC K100M (Hydroxypropyl methylcellulose) was obtained from F. D. Enterprise Corporation; ^2^ Chitosan Mw: 20–35 kDa from Shin Era Technology Co., Ltd.

**Table 2:** The viscosity and texture parameters of HCT hydrogel containing various concentrations of HPMC in comparison with a marketed product (ELYZOL^®^).

Formulation	Viscosity (cps)	Hardness (N)	Adhesiveness (N mm)	Compressibility (N mm)
TBO only ^a^	4.95 ± 0.37	0.13 ± 0.05	0.23 ± 0.03	0.56 ± 0.05
Mixture of TBO & chitosan ^b^	4.59 ± 0.28	0.14± 0.02	0.25 ± 0.03	0.78 ± 0.03
F-1	36.56 ± 1.50	0.18 ± 0.03	0.88 ± 0.03	1.46 ± 0.18
F-2	46.64 ± 2.13	0.22 ± 0.02	1.89 ± 0.07	2.67 ± 0.59
F-3	294.8 ± 20.6	0.56 ± 0.03	3.88 ± 0.12	5.33 ± 0.24
F-4	495.4 ± 58.9	0.93 ± 0.06	6.07 ± 0.21	8.16 ± 0.49
F-5	629.8 ± 113.6	1.67 ± 0.04	14.10 ± 0.33	13.89 ± 0.90
ELYZOL^® c^	187.4 ± 18.3	0.18 ± 0.02	0.95 ± 0.08	2.33 ± 0.49

^a^ 20 μM TBO solution; ^b^ 20 μM TBO combined with chitosan (0.25%, *w*/*w*) in 1% acetic acid; ^c^ ELYZOL^®^ is a topical antibiotic gel.

**Table 3:** Injectability of HCT hydrogel in various concentrations of HPMC in comparison with a marketed product (ELYZOL^®^).

Formulation	Injectability (gw·min^−1^)	
	200 (gw)	500 (gw)
distilled water	8.983 ± 0.682	15.909 ± 1.351
TBO only	8.053 ± 0.432	15.861 ± 1.642
Mixture of TBO & chitosan	7.241 ± 0.334	10.340 ± 1.031
F-1	2.808 ± 0.132	8.849 ± 0.872
F-2	0.151 ± 0.062	7.251 ± 0.703
F-3	0.008 ± 0.001	0.012 ± 0.004
F-4	0.0021 ± 0.002	0.0115 ± 0.003
F-5	N/A	0.0013 ± 0.001
ELYZOL^®^	0.324 ± 0.160	10.124 ± 1.095

N/A: Not available.

To evaluate the practical use in clinical application, we further quantify the injectability of this HCT hydrogel. A quantitative method for measuring the ease of injectability was performed by placing a fixed loading weight on the top of the plunger and the amount of hydrogel expelled from the syringe was then measured under one minute. As shown in Table 3, the decreased injectability was associated with the increase in HPMC concentration regardless the loading force. The F-5 HCT hydrogel containing 3% (*w*/*w*) HPMC was the most difficult one to be expelled from the syringe. On a comparative basis, the injectibiltiy of F-1 and F-2 were acceptable when compared to ELYZOL^®^ under 200 and 500 g loading mass, respectively.

The effect of increasing HPMC concentration in the formulation on the hardness, compressibility, and injectability of the gel also related to product viscoelasticity. Increased product viscoelasticity, particularly elasticity, offered an increased resistance to product deformation in texture and injectability, resulting in an increase in product hardness and work required for product compression/expulsion.

### 2.2. Assessment of PDI Efficacy of HTC Hydrogels against S. aureus and P. aeruginosa Biofilms

**Figure 1:**
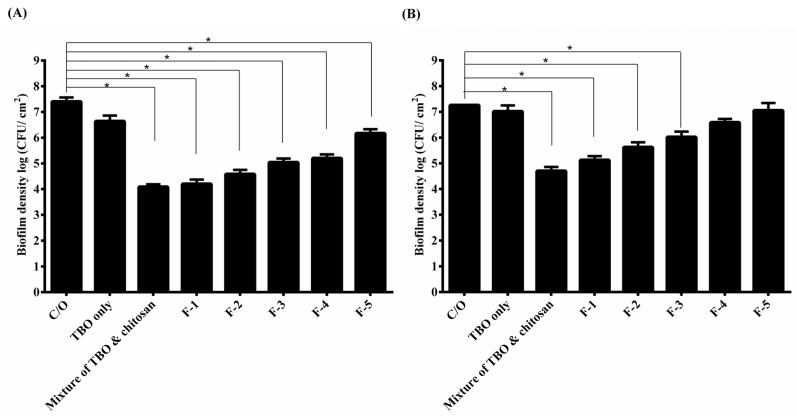
Cell survival fraction of *S. aureus* (**A**) and *P. aeruginosa* (**B**) biofilm treated with HCT hydrogels-mediated PDI. Biofilm cells were incubated with 20 μM TBO or HCT hydrogels containing 20 μM TBO for 1 h, followed by light exposure at 20 J·cm^−^^2^. Each value is the mean from three independent experiments ± standard deviation. * *p* < 0.05.

PDI efficacy of these HCT hydrogels was investigated against the biofilm cells of *S*. *aureus* and *P*. *aeruginosa*. As shown in Figure 1A, approximately 1-log_10_ reduction in viable counts was found in *S*. *aureus* biofilm cells incubated with TBO, while the mixture of TBO and chitosan showed a pronounced antimicrobial effect (~3.5-log reduction) under a light dose of 20 J·cm^−2^ (Figure 1A). The F-1 and F-2 HCT hydrogel also caused similar PDI efficacy as found in the mixture of TBO and chitosan. However, a significant decrease (*p* < 0.05) in the PDI efficacy was observed when the HPMC concentration increased to 1% (*w*/*w*) or above. The survival fraction approximately reduced from 3- to 1-log when treated with F-1 to F-5, respectively. These results indicated that higher concentration of HPMC could increase the viscosity of hydrogel, but might constrain TBO release and decrease the PDI efficacy of HTC hydrogel. Similar results were also found in *P. aeruginosa* biofilm treated with HCT hydrogel (Figure 1B). HCT hydrogels (F-1, F-2 and F-3) mediated PDI resulted in a 1- to 2-log bacterial killing in the biofilm cells of *P. aeruginosa* compared to 3-log killing treated with mixture of TBO and chitosan (Figure 1B). The lower PDI efficacy in *P. aeruginosa* is expected. Lipopolysaccharides are a major constituent of the *P*. *aeruginosa* membrane, and changes observed in membrane structure may result in changes to the drug permeability barrier [12].

### 2.3. Penetration of TBO into Biofilms

**Figure 2:**
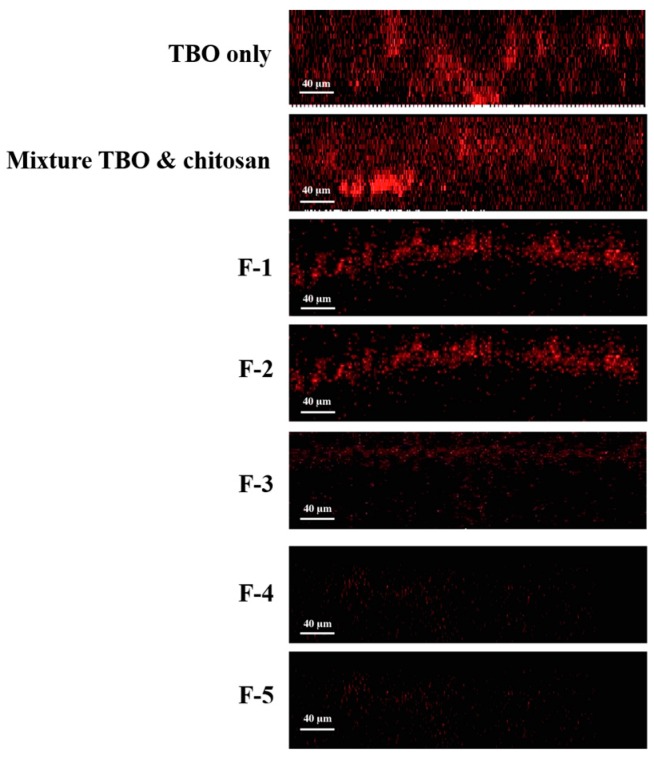
Confocal fluorescence imaging (X–Z) of *S*. *aureus* biofilms treated with HCT hydrogel for 1 h. The fluorescent signal from the TBO only biofilm was of higher intensity and extended deeper than 115 μm.

It has been shown that the drug release is diffusion-controlled and depends mostly on the viscosity of the hydrogel formed [13]. In addition, Ford *et al.* have reported that the drug release was slowly in high viscosity HPMC gelling agents [14,15]. To verify whether higher HPMC concentration constrains TBO release, confocal microscopy analysis was performed to characterize the penetration of TBO into viable *S*. *aureus* biofilms. Figure 2 shows the X–Z confocal images obtained from biofilms treated with TBO only, mixture of TBO and chitosan and HCT hydrogels containing different concentrations of HPMC, respectively. As expected, the overall fluorescence intensity and the penetration depth obtained from biofilm treated with TBO or the mixture of TBO and chitosan was significantly stronger than those of treated with HCT hydrogels.

The quantitative analysis as shown in Table 4 demonstrated a significant decrease of TBO in deeper layers of the biofilm treated with HTC hydrogels. In the group treated with TBO solution, the fluorescent signal extended to a depth of ~110 μm, whereas the signals only extended to a depth in a range of 10 to 83 µm in the specimen treated with HCT hydrogels. These results indicate that the increase in the HPMC concentration of HTC hydrogel indeed constrains TBO release into the deeper layer of *S. aureus* biofilm, which further explain the reduced PDI susceptibility of biofilms treated with hydrogel containing higher concentration of HPMC.

For clinical practice, PDI is more-easily carried out for localized infection. In this regards, a delivery system with good adhesion property is required to apply photosensitizer onto the infected tissues. Longer retention in the absorption site allows the photosensitizer to have a chance to bind and penetrate the biofilm. In this study, incorporation of HPMC offered a better adhesion property for this topical dosage form. However, optimizing HPMC concentration is necessary to obtain the optimized texture properties and PDI efficacy.

**Table 4:** The average distribution depth of TBO into the biofilm. Values are mean ± standard deviation.

Formulation	Average Depth (μm)
TBO only	>115 ± 15
Mixture of TBO & chitosan	110 ± 13
F-1	83 ± 9
F-2	52 ± 6
F-3	35 ± 4
F-4	18 ± 2
F-5	10 ± 2

### 2.4. Influence of Tilt Degree on PDI

**Figure 3:**
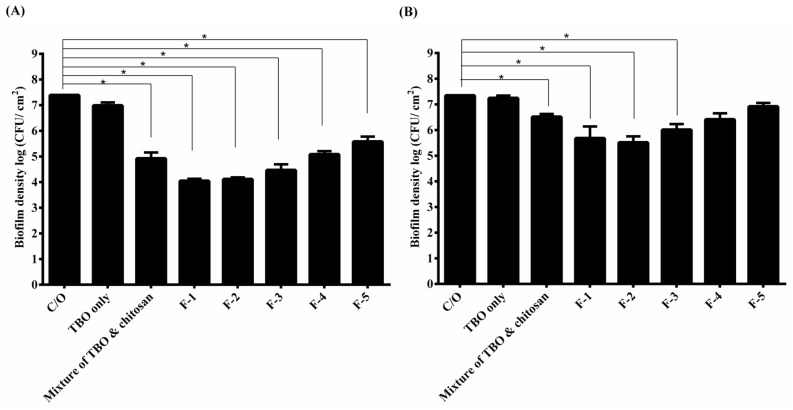
Cell survival fraction *S*. *aureus* (**A**) and *P*. *aeruginosa* (**B**) biofilm incubated with various formulas of HCT hydrogel for 1 h under 90-degree tilt, and then subjected to 20 J·cm^−2^ of light illumination. Each value is the mean from three independent experiments ± standard deviation. * *p* < 0.05.

Based on the texture analysis, we have shown that high viscosity of dosage forms might provide a better adhesion on the treatment areas and prolong the drug contact (Table 2). To mimic the clinical use, we tilted the plates containing biofilms and HCT hydrogel to 90 degrees for the following incubation, and then examined the PDI efficacy. Figure 3 shows the survival rate of *S*. *aureus* and *P*. *aeruginosa* biofilms with tilt. Interestingly, the results were quite different from those shown in Figure 1. Under tilt, there was no significant reduction in the survival rate of biofilm treated with the TBO. Treated with the mixture of TBO and chitosan approximately 2.5- and 0.7-log reductions were found against *S*. *aureus* and *P*. *aeruginosa* biofilm, respectively. Furthermore, F-1 and F-2 showed a significant better antimicrobial effect than the control against *S*. *aureus* and *P*. *aeruginosa* biofilm. Both approximately 3.5- and 2-log reductions in viable cell count were found against *S*. *aureus* and *P*. *aeruginosa* biofilm, respectively. The higher adhesive character contributed by increasing the incorporation of HPMC seems to provide a better contact with the biofilm and leading to a superior antimicrobial effect. However, the PDI efficacy decreased when the concentration of HPMC increased to 1% (*w*/*w*) or higher.

Antimicrobial PDI has provided new hope for controlling microbial infections. However, the area of infection might not be a plane or horizontal region. In this regards, to retain or prolong the drug delivery system on the absorption site becomes a key issue for a successful treatment. Incorporation of HPMC into HCT hydrogel can increase its adhesiveness and further warrant its therapeutic efficacy.

### 2.5. PDI Treatment Adjustment of HCT Hydrogel

**Figure 4:**
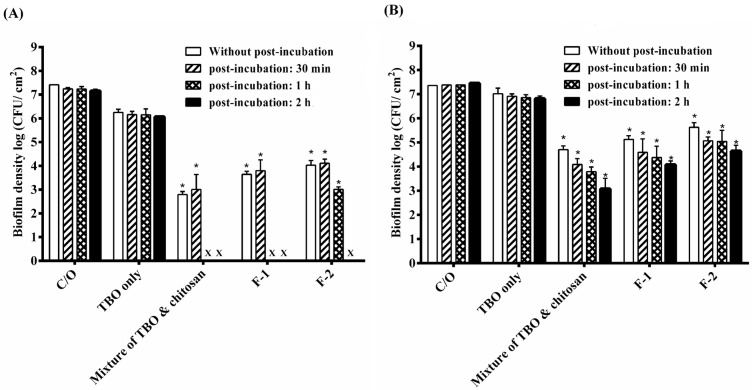
Cell survival fraction of *S*. *aureus* biofilm (**A**) and *P. aeruginosa* biofilm (**B**) after being incubated with various formulas of HCT hydrogel for 1 h and then subjected to 20 J·cm^−2^ of the red light illumination. After PDI, biofilm cell was maintained in HCT hydrogel for 0, 30 min, 1 h and 2 h and then plate count. Each value is the mean from three independent experiments ± standard deviation. * *p* < 0.05. x means no biofilm colonies were counted in this study.

Previously, we have shown that a 30-min incubation time with chitosan following PDI can potentiate the bactericidal efficacy in planktonic cells and biofilms [6]. This suggests that the potentiated effect of chitosan worked after the bacterial damage induced by PDI. Therefore, the PDI efficacy of the F-1 and F-2 HCT hydrogel was examined with an additional incubation after light irradiation. As shown in Figure 4A, after light irradiation, the complete inactivation against *S. aureus* biofilm was observed with an additional 1- or 2-h incubation in the group treated with F-1 and F-2 HCT hydrogel. Although a complete eradication was not found in *P*. *aeruginosa* biofilm, additional incubation after PDI could result in a significant bactericidal efficacy (Figure 4B).

In this study, after light irradiation, a longer retention time of HTC hydrogels on biofilms is required to enhance the bactericidal efficacy against *S. aureus* and *P. aeruginosa*. Meanwhile, the augmented bactericidal effect of chitosan in HTC hydrogel reduced as increasing the HPMC concentration. These results indicate that, like TBO, chitosan released from the HTC hydrogel was probably constrained due to the high viscosity network of hydrogel. Therefore, a longer retention time was required to achieve the augmented bactericidal efficacy.

### 2.6. In Vivo Study

**Figure 5:**
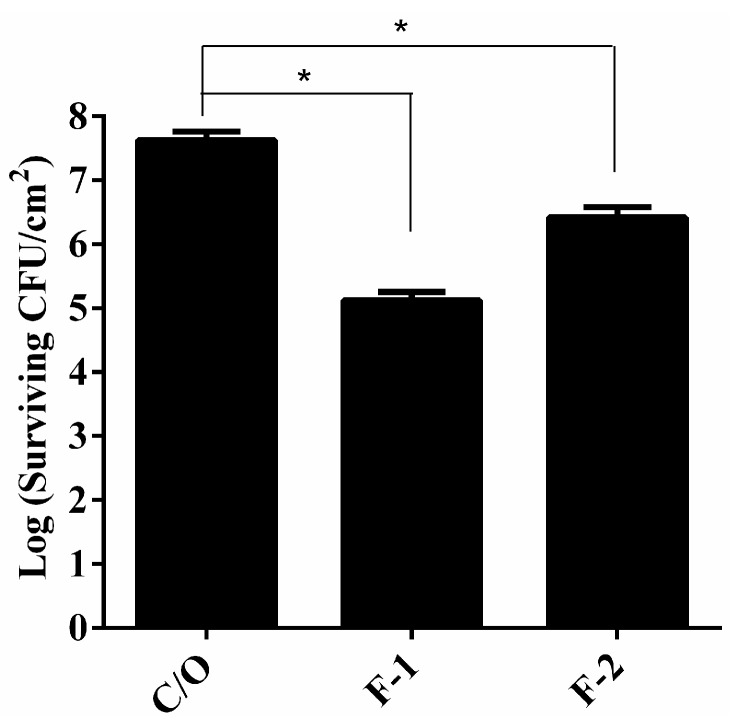
*S*. *aureus* viable counts in burn wounds incubated with PBS, F-1 or F-2 HCT hydrogels. After 1 h incubation, light dose of 100 J·cm^−2^ were applied to the burn wounds. After PDI, HCT hydrogel was maintained in the wounds for another 2 h incubation and then plate count. Reported values are the means values ± SD. * *p* < 0.05.

To examine the *in vivo* antimicrobial activity of HCT hydrogel, the PDI efficacy was verified in the animal model of rat skin burn wounds infected with *S*. *aureus*. These infected wounds were incubated with F-1 or F-2 HTC hydrogels for 1 h and then irradiated with 100 J·cm^−2^ of 630 nm laser light. Figure 5 demonstrates that the infected wounds treated with HCT hydrogel mediated PDI showed a significant reduction in bacterial cells survival compared to that of wounds treated with PBS. On the other hand, the overall reduction rate reduced as increasing the concentration of HPMC in HTC hydrogel. Although substantial reductions in the viable count of *S*. *aureus* biofilm in the wounds were achieved, the kills observed in this *in vivo* model were substantially lower than those reported in *in vitro* studies. Except for the biofilm formation, the reduced efficacy in the *in vivo* study might be due to the host tissue competing with the photosensitizer and chitosan released from hydrogel. In this regard, tissue damage might be induced. Based on the visualized observation, we did not find significant difference on the healing of rat tissues between the groups treated with PBS or HCT hydrogel. In the future, further pathological analysis is required to examine whether HCT hydrogel will cause tissue damage after light irradiation.

## 3. Experimental Section

### 3.1. Materials

Chitosan (*M*w 25 to 35 kDa) with degree of deacetylation >90% in powder form was purchased from Shin Era Technology (Taipei, Taiwan). Toluidine blue O (TBO) and acetic acid were obtained from Sigma-Aldrich (St. Louis, MO, USA). HPMC (Hydroxypropyl methylcellulose, K100M) was purchased from F. D. Enterprise Corporation. Microbial culture media Tryptic soy broth (TSB) and agar were purchased from Difco (Detroit, MI, USA).

### 3.2. Preparation of HCT Hydrogel

Various hydrogel formulations were prepared using HPMC, chitosan and TBO. Chitosan powder was dissolved in 1% aqueous acetic acid solution under magnetic stirring for 30 min at room temperature to make a 0.75% (*w*/*w*) chitosan solution. TBO (60 μM) was prepared in distilled water. An accurately weighed quantity of HPMC was stirred in 50 mL distilled water separately at different concentrations ranging from 0.75% to 9% (*w*/*w*). Various formulations were desired mixtures by 0.75% (*w*/*w*) chitosan/acetic acid solution, 60 μM TBO solution and HPMC solution (volume ratios of HPMC:chitosan:TBO = 1:1:1) and stirred at room temperature until completely dissolved. The hydrogels were left to swell for at least 24 h at room temperature before further experiments. A final concentration of 20 μM of TBO content was prepared for comparison between TBO only and HTC hydrogel formulations throughout the experimental period.

### 3.3. Viscosity and Texture Profile Analysis of HCT Hydrogel

The viscosity of HCT hydrogel (in 100 mL lots) was determined by using Brookfield viscometer (Brookfield Engineering Laboratories; Stoughton, MA, USA). The spindle number six was dipped in the HCT hydrogel and rotated at 3, 10, 30, and 50 rpm at room temperature. The viscosity of hydrogel was determined by the viscometer.

Texture profile analysis (TPA) of pharmaceutical hydrogels was performed using a TA-XT2 Texture Analyzer (Stable Micro Systems, Surrey, London, UK) in texture profile analysis mode, as previously described [16,17]. The specimens were lubricated with mineral oil on both ends prior to measurement, and all measurements were made on hydrogels equilibrated to ambient temperature. In brief, HCT hydrogels were packed to a fixed height into a 10 mL McCartney bottle. In TPA, an analytical probe with 10 mm diameter was compressed twice into each sample at a defined rate of (2.0 mm·s^−1^) and to a defined depth of 15 mm. A delay period of 15 s was allowed between the end of the first and beginning of the second compressions. The measurements were performed in quintuplicate, where each sample was measured five times. The hardness, adhesiveness, and compressibility of HCT hydrogels were determined from the resultant force-time plot. In addition, the viscosity and texture profile of HTC hydrogels were further compared with a marketed formulation. A topical antibiotic gel (ELYZOL^®^), composed of metronidazole, used for treating bacterial infections in the mouth. It was selected as the reference for optimizing our formula suitable for clinical use.

### 3.4. Assessment of the Injectability

The injectability of the HCT hydrogel was evaluated with a device according to a previously reported method [8]. Briefly, 5 mL HCT hydrogel was filled into 5 mL plastic syringe. A 23G × 1.25 inch needle was fixed on the syringe. 200 and 500 g mass of weight was then mounted vertically on the top of the plunger, respectively and the weight of the HCT hydrogel expelled from the syringe in one minute was recorded. Each formulation including a marketed product (ELYZOL^®^) was performed six times and the average weight was calculated.

### 3.5. Biofilm Preparation

*Staphylococcus* *aureus* (*S*. *aureus*, BCRC 10780) was purchased from the Bioresource Collection and Research Center (Hsinchu, Taiwan). *Pseudomonas* *aeruginosa* (*P*. *aeruginosa*, ATCC 27853) was purchased from ATCC (Manassas, VA, USA). TSB was used as the liquid medium for *S*. *aureus* and *P*. *aeruginosa*. A 0.1% medium was used for batch cultures, whereas 0.01% medium was used for continuous biofilm cultures.

Bacterial biofilms were cultured on a rotating disk reactor modified from the design of Pitts *et al.* [18]. Briefly, the reactor consisted of a 500 mL polypropylene container, a Teflon rotor, and 24 removable 316L stainless steel disks (0.6 cm in diameter) inserted on the rotor onto which the biofilm are culture. One milliliter of each bacterial suspension seed culture was inoculated into 150 mL of 0.1 × TSB in the reactor and incubated at room temperature overnight. After incubation, the reactor was continuously fed with 0.01 × TSB at a rate of 240 mL·h^−1^. The Teflon rotor was embedded with a magnetic stir bar on the bottom and was driven by a stirrer at 60 rpm for *P. aeruginosa* biofilm. The biofilms reached a steady state after 24 h, and the average biofilm density was 5 × 10^7^ CFU·cm^−2^.

### 3.6. Confocal Scanning Laser Microscopy (CSLM)

To accommodate for the width of the confocal microscope objective, biofilms were grown on 1.5 cm high TSB-agar medium for two days in transwell plates anaerobically as described above. For optimum biofilm development, the plaque/TSB inoculum contained 10^9^ CFU·mL^−1^. A Leica SP2 confocal scanning fluorescence microscope (Leica Inc.: Malvern, PA, USA) equipped with a 20× or 40× water-dipping objective lens was employed to observe the fluorescence emission of TBO. An argon laser (635 nm) was used as the excitation light source of TBO. Sections were collected at various intervals and were then analyzed by image-processing techniques to assess the distribution of TBO within the biofilm matrices.

### 3.7. HCT Hydrogel Mediated PDI in Biofilm Cells

The disks with biofilms were placed in a sterile 48-well multititer plate. First, each biofilm treated with 350 μL of HCT hydrogel in the dark for 1 h, respectively, and then moved to a new dish. The disks were irradiated with the LED array, with the wavelength centered at 635 nm with a full-width-at-half-maximum bandwidth of 20 nm. After light irradiation, the disks continued to incubate for different periods of time in the dark. Then, the disk with biofilms was put into test tubes containing 10 mL sterile phosphate-buffered saline and vigorously vortexed to remove the biofilm from the disks. The resulting bacterial suspensions were diluted and plated on TSB agar, and the colonies formed after 18 h of incubation at 37 °C were counted. The number of viable cells was determined by averaging the CFU on three plates as described in Section 3.8.

### 3.8. Bacterial Cell Survival Assay

The CFU of a bacterial suspension was counted using the following standard protocol. Aliquots (10 μL) of appropriate dilutions (from 10^−1^ to 10^−6^) were plated on TSB agar plates and incubated at 37 °C in the dark for 18 h. The surviving fraction was calculated as N_PDI_/N_0_, where N_PDI_ is the CFU·cm^−2^ after antimicrobial photodynamic inactivation and N_0_ is the CFU·cm^−2^ in the initial sample. The dark toxicity of the substrates, defined as the intrinsic toxicity of the compounds in the absence of light, was monitored by evaluating the surviving fraction of non-illuminated bacterial samples and calculated as N_DARK_/N_0_, where N_DARK_ is the CFU·cm^−2^ of the non-illuminated samples.

### 3.9. Rat Model of Skin Burn Wound Infected S. aureus

Three adult male Wistar rats (8–10 month old), 500–650 g, were anesthetized by intraperitoneal injection of Ketamine (50 mg·kg^−1^) plus Rompun (5 mg·kg^−1^). The dorsum of the rat was shaved with hair clippers and a depilatory (Nair, California City, CA, USA). One rat was subjected to four wounds with third degree burn by pressing four 1 cm × 1 cm copper plates preheated to 120 °C against its back for 12 s [19]. To facilitate bacterial infection, the stratum corneum of the burned skin was peeled off with a scalpel, because stratum corneum consists of anuclear cells that are replete with the tough protein keratin and hydrophobic lipids that waterproof the skin. The wound area was protected using a PE foam sheet with a 1 cm × 1 cm defect in the middle, which was tightly adhered to skin using tissue glue. This created a well surrounding the wound to prevent applied liquid from escaping.

Burn wounds were infected by the smearing of 20 μL of the concentrated *S. aureus* suspension containing 10^6^ cells onto the burned surfaces. After 2 h post-infection, HCT hydrogel (200 μM TBO) was applied to the wound and incubated for 1 h. Then, each infection area was irradiated for 100 J·cm^−^^2^ by a homemade 630 nm diode laser. After irradiation, HCT hydrogel was continued to incubate for 2 h. Finally, tissue biopsies were removed from the wound. The sampled tissues were weighed and homogenized in PBS. Viable cell counts were performed and standardized per gram of tissue biopsies. All the surgical and experimental procedures were approved by Institutional Animal Care and Use Committee of National Taiwan University College of Medicine and College of Public Health and were in accordance with the guidelines of the National Science Council of Taiwan.

### 3.10. Statistics Analysis

All experiments were repeated three times. Results are expressed as the mean ± standard deviation. Differences between two means were assessed for significance by the two-tailed Student *t*-test, and a *p* value of <0.05 was considered significant.

## 4. Conclusions

The optimal choice of bioadhesive formulation for use in topical antimicrobial PDI will involve a compromise between achieving the necessary release rate of drug and the mechanical characteristics of the formulation, as these factors will affect clinical efficacy and the ease of topical application. TBO biofilm penetration relates to the physicochemical properties of HTC hydrogel. The enhancement strategies for TBO diffusion into biofilm together with formulation strategies should be considered in detail for future clinical applications.
